# BsRADseq: screening DNA methylation in natural populations of non‐model species

**DOI:** 10.1111/mec.13550

**Published:** 2016-03-28

**Authors:** Emiliano Trucchi, Anna B. Mazzarella, Gregor D. Gilfillan, Maria T. Lorenzo, Peter Schönswetter, Ovidiu Paun

**Affiliations:** ^1^ Department of Botany and Biodiversity ResearchUniversity of ViennaRennweg 141030ViennaAustria; ^2^ Department of BiosciencesCentre for Ecological and Evolutionary SynthesisUniversity of OsloPO Box 1066 BlindernN‐0316OsloNorway; ^3^ Department of Medical GeneticsOslo University HospitalPO Box 4590 NydalenN‐0420OsloNorway; ^4^ Institute of BotanyUniversity of InnsbruckSternwartestrasse 15A‐6020InnsbruckAustria

**Keywords:** bisulfite sequencing, bsRADseq, DNA methylation, population epigenetics, RADseq

## Abstract

Epigenetic modifications are expected to occur at a much faster rate than genetic mutations, potentially causing isolated populations to stochastically drift apart, or if they are subjected to different selective regimes, to directionally diverge. A high level of genome‐wide epigenetic divergence between individuals occupying distinct habitats is therefore predicted. Here, we introduce bisulfite‐converted restriction site associated DNA sequencing (bsRADseq), an approach to quantify the level of DNA methylation differentiation across multiple individuals. This reduced representation method is flexible in the extent of DNA sequence interrogated. We showcase its applicability in three natural systems, each comprising individuals adapted to divergent environments: a diploid plant (*Heliosperma,* Caryophyllaceae), a tetraploid plant (*Dactylorhiza,* Orchidaceae) and an animal (*Gasterosteusaculeatus,* Gasterosteidae). We present a robust bioinformatic pipeline, combining tools for RAD locus assembly, SNP calling, bisulfite‐converted read mapping and DNA methylation calling to analyse bsRADseq data with or without a reference genome. Importantly, our approach accurately distinguishes between SNPs and methylation polymorphism (SMPs). Although DNA methylation frequency between different positions of a genome varies widely, we find a surprisingly high consistency in the methylation profile between individuals thriving in divergent ecological conditions, particularly in *Heliosperma*. This constitutive stability points to significant molecular or developmental constraints acting on DNA methylation variation. Altogether, by combining the flexibility of RADseq with the accuracy of bisulfite sequencing in quantifying DNA methylation, the bsRADseq methodology and our bioinformatic pipeline open up the opportunity for genome‐wide epigenetic investigations of evolutionary and ecological relevance in non‐model species, independent of their genomic features.

## Introduction

Many studies have documented epigenetic variation between individuals, particularly in plants (e.g. Cubas *et al*. 1999; Manning *et al*. 2006; Lira‐Medeiros *et al*. 2010; Paun *et al*. 2010; Schmitz *et al*. 2013; Alonso *et al*. 2014; Cortijo *et al*. 2014; Medrano *et al*. 2014; Dubin *et al*. 2015; Platt *et al*. 2015; Preite *et al*. 2015), also in animals (e.g. Liu *et al*. 2012; Chalker *et al*. 2013; Smith *et al*. 2015), and humans (e.g. Heyn *et al*. 2013). Three main causes of epigenetic variation among individuals have been proposed: (i) genetic variation with secondary epigenetic effects (Richards 2008); (ii) epigenetic instability, or spontaneous epimutations (including reversals) with rates several orders of magnitude higher than that of genetic mutations (Tal *et al*. 2010; Becker *et al*. 2011; Schmitz & Zhang 2011); and (iii) environmental sensitivity of epigenetic marks, or stochastic epimutations induced by environmental stress (Angers *et al*. 2010; Verhoeven *et al*. 2010; Dowen *et al*. 2012). These epigenetic changes can alter the expression of genes (e.g. Rapp & Wendel 2005; Li *et al*. 2012; Le *et al*. 2015), sometimes with important phenotypic consequences (Dowen *et al*. 2012; Ou *et al*. 2012; Latzel *et al*. 2013). In addition, these changes can be more permanent than plasticity, potentially affecting future generations (e.g. Jablonka & Raz 2009; Verhoeven *et al*. 2010; Daxinger & Whitelaw 2012; Ou *et al*. 2012; Silveira *et al*. 2013), and it has been shown that phenotypic variation caused by epigenetic differences can be stably inherited over several generations (e.g. Johannes *et al*. 2009). Indeed, recent studies have pointed to an important role of epigenetic variation in ecological processes (Bossdorf & Zhang 2011; Latzel *et al*. 2013). However, little information is available on the extent of epigenetic variation outside of model organisms, particularly in natural populations.

The most commonly studied epigenetic mechanism is DNA methylation, a biochemical addition of a methyl group to cytosine possible in three different sequence contexts: CG (also called CpG), CHG and CHH, where H represents any nucleotide except guanine (Jones & Takai 2001; Fulnecek *et al*. 2002). Methylations in each of these contexts have different molecular functions and are established by distinct DNA methylation pathways (Law & Jacobsen 2010). As a symmetrical context, CG methylation is maintained during DNA replication by the MET1 methyltransferase in plants and the DNMT1 in animals (Law & Jacobsen 2010). CG methylation is generally linked to gene body methylation, but it is also enriched around transposable elements (TEs; Lister *et al*. 2008). In vertebrates, but also in a few plants, an enrichment of unmethylated CG dinucleotides is specific for genomic regions containing sites of transcription initiation, within dense CpG islands (Feng *et al*. 2010a; Deaton & Bird 2011). Methylation in non‐CG context is more abundant in plants and it is predominantly encountered around TEs (Feng *et al*. 2010a). However, it has recently been shown that non‐CG methylation is also found in bacteria, fungi and some animal tissues; and may drive tissue‐specific functions (Schultz *et al*. 2015). In plants, CHG methylation is controlled by H3K9 histone methylation through the methyltransferase CMT3, whereas CHH methylation is guided by small RNAs through the DRM2 methyltransferase (Law & Jacobsen 2010). The important role of methyltransferase CMT2 in mediating non‐CG methylation has recently been described (Zemach *et al*. 2013; Stroud *et al*. 2014). In addition to the peculiarities of each cytosine context, DNA methylation can be site‐specifically regulated (Stroud *et al*. 2013), hence a great deal of methylation variation is expected across cell types, genotypes and environments.

There are several ways to quantify the diversity in DNA methylation between individuals (e.g. Schmitz & Zhang 2011), but only a few have been extensively used. Bisulfite sequencing, i.e. sequencing DNA previously treated with sodium bisulfite, which causes deamination of unmethylated cytosines to uracil (Frommer *et al*. 1992; Henderson *et al*. 2010; Laird 2010), has been the source of much data on methylation patterns, either for specific loci, or coupled with whole‐genome sequencing (Schmitz & Zhang 2011). Traditionally, a modified amplified fragment length polymorphism (AFLP) technique has been used to obtain information on DNA methylation patterns by comparing fingerprints produced with isoschizomers (enzymes that recognize the same restriction site but differ in methylation sensitivity) via methylation‐sensitive or secondary‐digest AFLP (MSAP or SD‐AFLP; Baurens *et al*. 2003; Schrey *et al*. 2013). This technique has been particularly appealing for ecological and evolutionary studies because it is neither as cost prohibitive nor as computationally demanding as genomic approaches, and thus allows for population‐level analyses. However, aside from AFLP‐inherent problems (i.e. the anonymous nature of the fragment length markers is potentially affected by homoplasy; the low information content of the dominant markers coupled with methylation resolution only at restriction sites), the MSAP method also suffers from the homogenizing effect of two PCR rounds, essentially transforming quantitative DNA methylation rates into binary presence─absence information (Schrey *et al*. 2013).

Here, we present a high‐resolution method for quantification of DNA methylation, bsRADseq, which combines restriction site associated DNA sequencing (RADseq; (Baird *et al*. 2008; Etter *et al*. 2011) with bisulfite sequencing. This NGS‐based technique results in a reduced representation of the genome that is consistent across multiple individuals, revealing the patterns of DNA methylation at base‐pair resolution across the fragments represented. If a reference genome is available, the sequences can be mapped back to known positions. However, for non‐model organisms without a published genome, we describe a method for the construction of synthetic references for mapping bisulfite‐converted RADseq reads using loci constructed from standard RADseq data. BsRADseq has the same flexibility as RADseq (Etter *et al*. 2011), where the choice of the restriction enzyme determines the proportion of the genome that will be genotyped. In addition, bsRADseq features all standard characteristics of bisulfite sequencing, including accuracy and base‐pair resolution when detecting DNA methylation (Schmitz & Zhang 2011). This technique opens up the opportunity for genome‐wide epigenetic investigations of evolutionary and ecological relevance, especially for questions that require information from a large number of individuals in species with a medium to large genome size. We aim here to demonstrate the flexibility of this approach across a range of model and non‐model organisms and to investigate the extent of epigenetic divergence between individuals that thrive in different ecological conditions.

## Materials and methods

### Study systems

We exemplify the potential of bsRADseq by producing quantitative estimates of divergence in DNA methylation patterns between individuals that thrive in different habitats in three systems, one animal and two plants, including one polyploid.

The first system is the model organism three‐spine stickleback (*Gasterosteusaculeatus,* Gasterosteidae). This fish has been extensively used in biological research, mainly due to an adaptive radiation that happened 10–12 000 years ago. During this radiation, hundreds of freshwater lakes in the northern hemisphere were independently colonized from the large panmictic saltwater population present in the ocean (Bell & Foster 1994; Foster & Baker 2004). The resulting populations are particularly well suited to study questions of local adaptation and parallel evolution. With this work, we show that it is feasible to add epigenomic data to the well‐established genomic information (Colosimo *et al*. 2005; Jones *et al*. 2012; Roesti *et al*. 2012) by using a flexible and cost‐effective approach that expands our screening beyond GC‐rich islands (cf. Smith *et al*. 2015).

We also used our approach to investigate the difference in DNA methylation between two closely related species from *Heliosperma pusillum* sensu lato (Caryophyllaceae)*. Heliosperma pusillum* sensu stricto is found in humid rocky, alpine habitats, whereas the small populations of its close relative *H. veselskyi* can be found only below the tree line, in dry habitats such as below overhanging rocks (Neumayer 1923). The taxa are morphologically distinct; *H. veselskyi*is covered by a dense indumentum, whereas *H. pusillum*is glabrous. In spite of having been described as separate species, they are still fully interfertile (C. Bertel, B. Frajman & P. Schönswetter, unpublished). In addition, molecular investigations supported a history of multiple independent origins of the population of one ecotype (likely *H. veselskyi*) from the other (Frajman & Oxelman 2007; Frajman *et al*. 2009; E. Trucchi, B. Frajman, T. Haverkamp, P. Schönswetter & O. Paun, unpublished).

Lastly, we applied bsRADseq to a system of allopolyploids in the orchid genus *Dactylorhiza*. We compared two sibling tetraploid species, *D. majalis* and *D. traunsteineri*, that are ecologically and morphologically distinct (Paun *et al*. 2010) and resulted from independent, but unidirectional hybridization events between the same diploid parents. Whole‐genome doubling and hybridization induce major genomic and transcriptomic responses, including epigenetic alterations of parental homeologs, which can affect the ecological properties of the resulting polyploids (Madlung & Wendel 2013). Earlier results from MSAP suggested a clear differentiation between the methylation profiles of these tetraploid *Dactylorhiza* species (Paun *et al*. 2010). Here, we test if a clear differentiation between these species is still detectable using our approach.

### DNA samples

In our three bsRADseq proof‐of‐principle tests, we used DNA purified using standard extraction protocols (DNeasy kit for animal or plant tissue from Qiagen). Specifically, we used DNA from four stickleback fish, two individuals from a marine population sampled near Bergen, Norway, and two individuals sampled in the freshwater lake Mosvatn, close to Stavanger, Norway (Table S1, Supporting information). For the *Heliosperma* experiment, we used nine individuals from each of two neighbouring locations, an alpine population of *H. pusillum* and a montane population of *H. veselskyi* from Val Gardena in the Italian Dolomites (Table S1, Supporting information). In the case of tetraploid *Dactylorhiza,* we were interested in unravelling epigenetic differentiation between the species that exceeds within‐species differentiation, so we analysed individuals from two different geographical regions for each of the two species of *Dactylorhiza*. We used two individuals of *D. majalis*, one from Lanskrona, Sweden, and one from Mooshuben, Austria, and two individuals of *D. traunsteineri*, one from Lojsthajd, Gotland, Sweden, and one from the North Uistisland, United Kingdom (Table S1, Supporting information).

### Library preparation

**Figure 1:**
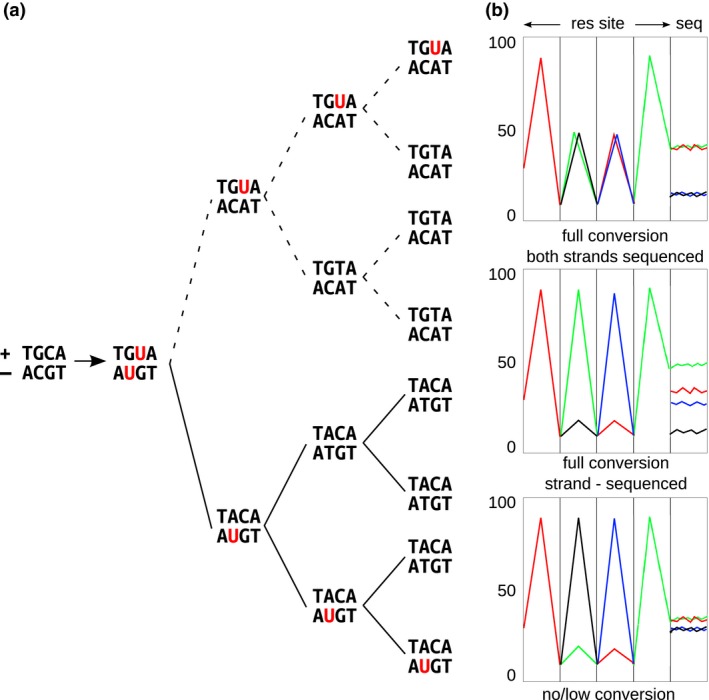
Sketch of directional bisulfite conversion used in bsRADseq: (a) Nonmethylated cytosines in the overhanging portion of the restriction site left by SbfI are converted by bisulfite treatment on both strands, ‘+’ and ‘−‘ (red). The converted ‘−‘ strand serves as template during RADseq directional amplification. Sequencing of the complementary to converted ‘−‘ strand is then carried out. (b) Three of the possible sequencing products, showing the bases of the restriction site (res site) and the expected average base composition in the remaining positions over all reads (seq): full bisulfite conversion of cytosines and sequencing of both strands (upper panel); full bisulfite conversion of cytosines but sequencing of the ‘−‘ strand only (middle panel); no/low bisulfite conversion of cytosines and sequencing of both strands (lower panel). The middle panel outcome was observed in our bisulfite conversion and sequencing experiments. Proportion of reads containing the specific nucleotide is shown on the *y*‐axis. Standard color code (thymine: red, cytosine: blue, adenine: green, guanine: black) is used.

The bisulfite‐converted RAD sequencing libraries were prepared with SbfI, modifying some important aspects of the standard protocol for RAD sequencing described in Baird *et al*. (2008). Conversion of nonmethylated Cs into Us was performed after ligation of the P2 adapter and before the PCR enrichment step by treating the library with sodium bisulfite using a Cells‐to‐CpG Bisulfite Conversion Kit (Applied Biosystems). The following PCR amplification using KAPA HiFi HotStart Uracil+ MasterMix (Kapa) converted all Us into Ts. All individuals were tagged by unique six base‐pair combinatorial barcodes included in the P1 and P2 adapters, which allow for an individual‐based analysis. The combinatorial barcoding system significantly decreased the cost of methylated adapters. We employed a set of methylated P1 and P2 barcoded adapters to protect their sequence from modification during bisulfite conversion. In order to keep the cost of the methylated adapters low, a short version of the P1 sequences was chosen (sequence for the top oligo: 5′‐ACACTCTTTCCCTACACGACGCTCTTCCGATCTXXXXXTGCA; XXXXX denotes the barcode sequences; source: https://www.wiki.ed.ac.uk/display/RADSequencing/Home). A standard, long version of the P2 adapter was used. The short version of the P1 adapter was extended during the PCR step to the full length necessary to ligate the ILLUMINA flow‐cell using these primer pairs: forward primer 5′‐AATGATACGGCGACCACCGAGATCTACACTCTTTCCCTACACGAC and reverse primer 5′‐CAAGCAGAAGACGGCATACGA. Our version of the forward primer corresponds to the pre‐truseq version of ILLUMINA primer (PE PCR Primer 1.0), but without the last 13 nucleotides. This modification was deemed necessary because the last 13 bases of the forward primer are complementary to a portion of the P2 adapter sequences, which would cause improper annealing during PCR, and P2 to P1 conversion. Without this modification, P1─P1 ligated fragments clustered on the ILLUMINA flow‐cell and were sequenced, resulting in ~30% sequencing yield loss in preliminary experiments. The cytosine in the 5′‐TGCA‐3′‐overhang of the P1 adapter, matching the SbfI restriction site after cleavage, was left nonmethylated. That particular cytosine is in a CHG context as the full SbfI restriction site is CCTGCA/GG. Methylation in this context is expected to occur at very few positions in animals, and around 5% in plant species, with extreme cases of up to 20% (Feng *et al*. 2010a). As such, after bisulfite conversion and sequencing, the sequence of the overhanging restriction site is, in most cases, expected to be TGTAGG when the converted top strand is sequenced, and TACAAA when the complement to the converted bottom strand is sequenced (Fig. 1). The PCR enrichment step in the bsRADseq protocol, carried out with a forward primer on the P1 adapter, and a reverse primer on the complement to the divergent end of the Y‐shaped P2 adapter (Baird *et al*. 2008), typically results in sequencing mainly the newly synthesized complement to the bottom strand, and a minimum amount of original top strand (with respect to the P1 adapter sequence). Therefore, we expected mostly reads starting with the sequence TACAAA (Fig. 1). This strand‐specific sequencing in the RAD protocol is not producing any bias in scoring symmetrical methylation at CpG and CHG positions because the methylation information is normally replicated on both strands. On the other hand, methylation at (asymmetrical) CHH position on the bottom strand should also be detected with our approach. The libraries were sequenced on an Illumina HiSeq at CSF Vienna (http://csf.ac.at/ngs/) as 100 bp paired‐end reads.

### Analysis of bisulfite‐converted reads

After sequencing, the output file was de‐multiplexed using the script *process_radtags.pl* in the package stacks version 1.19 (Catchen *et al*. 2013) assigning reads to each individual on the basis of the barcoding sequences. Quality filtering was left as default (i.e. when the average quality score per base in any window of 15% of the read length dropped below 10, the read was discarded), but restriction site check at the beginning of the reads was disabled. This last setting was chosen in order to retain all reads with barcodes and directly analyse the bisulfite‐converted sequence of the restriction site. Because our barcodes differed from each other by at least three base pairs (barcode sequences were fully methylated), standard rescuing of barcode sequences with one sequencing error was still possible. We adopted two different strategies to score individual methylation and to compare it between relevant groups, depending on the availability of a reference genome.

### Analysis with a reference genome

In the case of the stickleback data set, a reference genome (ensembl, database release 72) was available. Converted reads of each of the four samples were separately mapped to this reference using the mapping routine available in the package bismark (Krueger & Andrews 2011). This mapping routine, whose core aligner can be bowtie (Langmead *et al*. 2009) or bowtie2 (Langmead & Salzberg 2012), is specifically designed for dealing with bisulfite‐converted reads. Instead of building one index of the reference genome to be used in the mapping step, the *Bismark_genome_preparation* routine builds two sets of indexes taking into account all possible conversions due to the sodium bisulfite treatment (C → T, or G → A on the complementary strand). Reads are then mapped using both of these indexes and the results are summarized into *sam* files. We tried both bowtie allowing up to three mismatches (option ‘‐n 3’) and bowtie2 allowing even more differences between the query reads and the reference sequence (option ‘–score_min L,0,‐0.6’) without appreciable differences in the results. Initially, the mapping was performed in a nondirectional modus (option ‘–non‐directional’), although our converted reads were expected to be from one strand only, in order to directly check the accuracy of mapping results. After mapping, we checked the summary report for each individual, recording the mapping efficiency, the number of cytosines screened, the distribution among the different contexts (CpG, CHG and CHH) and the differential representation of original strands vs. complementary to original strands. When mapping to a reference genome, the top or bottom strand specification (i.e., ‘+’ or ‘−‘) refers to the direction in the reference genome and not in the query reads. In this case, we expected mainly reads complementary to either the top or the bottom strand of the reference genome due to peculiarities of the RAD protocol (see above). After confirming this expectation in the summary reports for each sample, we proceeded to the following step of methylation calling. First, we ran the *Bismark_methylation_extractor* routine, ignoring the first four nucleotides in the reads (corresponding to the P1 adapter overhang), merging all non‐CpG cytosines, and producing one report per strand (or four in total: original top, original bottom, complementary‐to‐original top, complementary‐to‐original bottom). Then, we ran the *Bismark2bedgraph* routine, taking into account the methylation report files for complementary‐to‐original top and complementary‐to‐original bottom only. Although some of our reads (between 0.1% and 5%) could be recognized as the original top or original bottom, we decided to exclude them due to the low coverage. The final routine (*Bismark2bedgraph*) produced one summary report per chromosome in each sample including the coverage (read depth) and CpG methylation level in each position of the reference genome. These files were then processed as described below in the ‘Summary statistics of methylation pattern’ section.

### Analysis without a reference genome

**Figure 2:**
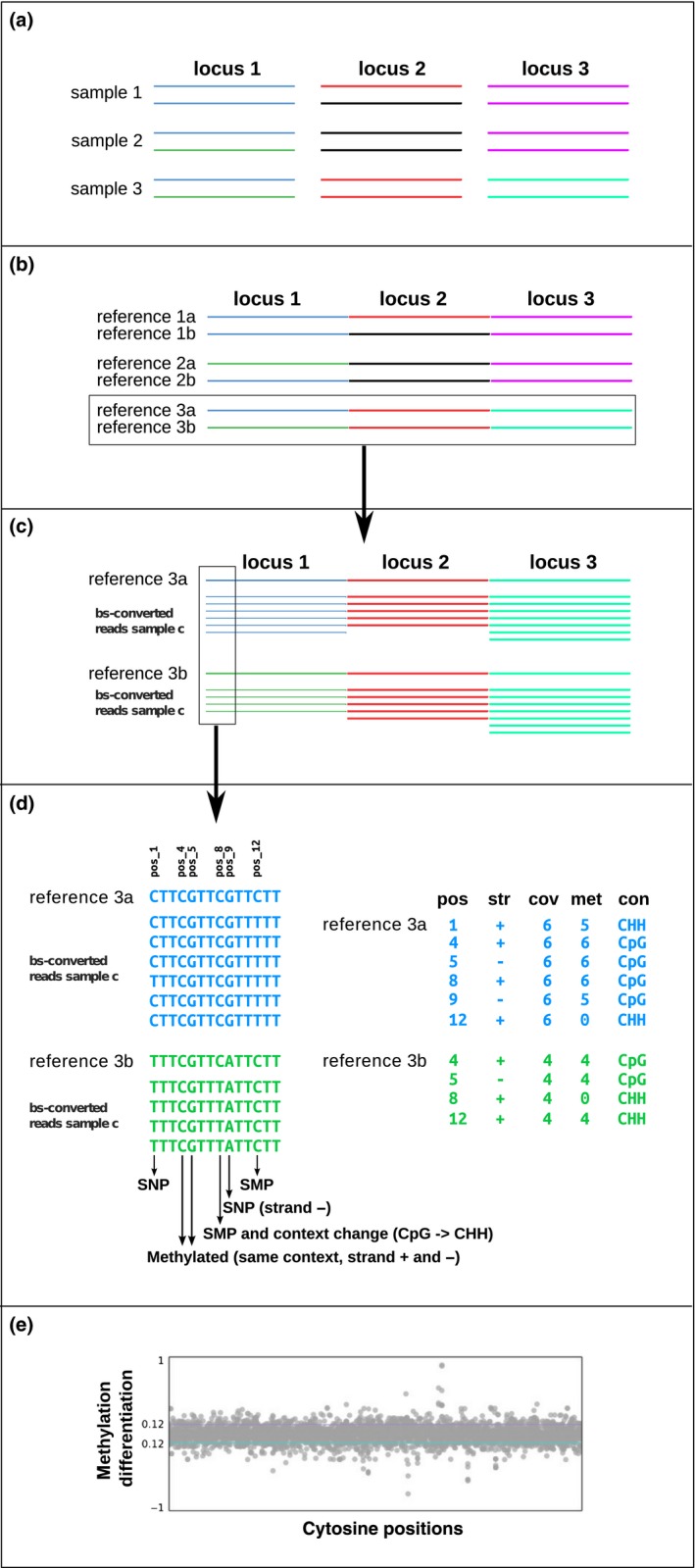
Workflow of the bsRADseq analysis when no reference genome is available. (a) Standard RAD sequencing data set assembled using stacks and unconverted reads (three hypothetical loci, 100 bp long, are exemplified and the different alleles per locus are indicated with different colours. (b) Individual reduced reference genome built using custom python scripts. (c) Mapping the converted reads of each individual sample to its own reduced reference genome using bismark and bowtie. (d) Analysis of the methylation level at each cytosine position separately in each individual sample using bismark. On the left: reads aligned to each reference, 14 base pairs shown; on the right, output of the *Bismark_methylation_extractor* function for the same base pairs. Pos: position; str: strand; cov: number of reads supporting that position; met: number of reads supporting methylation at that position; cont: context of the cytosine position according to the reference. (e) Results across individual samples are summarized using custom python scripts and statistical tests assessing significance of methylation differentiation between groups of samples can be performed. Here, the average methylation difference between the two *Heliosperma* species across all CHH positions screened is shown.

As no reference genome is available for the two plant systems analysed here, an initial step was required, where reduced‐representation substitute ‘reference genomes’ were built for *Heliosperma* and *Dactylorhiza* taking advantage of standard (non bisulfite‐converted) RADseq markers. Therefore, for each library including samples from these species, we separated an aliquot before the treatment with sodium bisulfite that was then directly amplified by PCR as a standard RADseq library, quality checked, and sequenced on an ILLUMINA HiSeq2500. After de‐multiplexing and quality filtering using standard options in *process_radtags* from the stacks package (Catchen *et al*. 2013), a catalog of loci for each of the two plant experiments was produced using the pipeline *denovo_map.pl* from the stacks package. A minimum number of 100 and 20 identical reads were selected in order to call a stack in *Heliosperma* and *Dactylorhiza* respectively. Up to seven mismatches were allowed to merge two stacks in a locus (‘‐M 7’) and up to nine mismatches were allowed to merge loci from different individuals (‘‐n 9’) *. *These values for the maximum number of mismatches were initially applied to take into account a putative high level of genetic diversity in each of the two species complexes. Downstream filtering (i.e. maximum ten polymorphic positions allowed per locus, maximum 0.75 heterozygosity allowed) was applied to minimize the probability of pooling paralogs in the same locus in the (genetic) reference. Calling haplotypes from secondary reads was disabled (‘‐H’ option; see the *Stacks* manual for the definition of primary and secondary reads). Given the presence of four tetraploid individuals in the *Dactylorhiza* experiment, four alleles were allowed at each locus with the option ‘ustacks:–max_locus_stacks 4’. Each catalog was then filtered for loci including between 0 and 10 SNPs and exported as a table with the script *extract_sql.pl* in the stacks package. They consisted of 1710 and 3180 loci for *Heliosperma* and *Dactylorhiza,* respectively. At this stage, we randomly selected a maximum of two alleles per locus in the *Dactylorhiza* catalog to simplify downstream analyses. Each catalog of loci was processed separately as follows: for each individual sample, we extracted the allele (or the two alleles, for heterozygous loci) for all loci in the catalog (Fig. 2a). These sequences were concatenated into two reduced reference genomes, choosing one or the other allele in heterozygous loci and duplicating the allele for homozygous loci (Fig. 2b). The choice of which allele in heterozygous loci would be in which concatenated reference sequence was made at random disregarding phasing, given that all loci should be well‐spaced along the genome due to the low cutting frequency of the restriction enzyme used in the library preparation step. This step resulted in two reduced‐representation genome references per individual sample that were used for mapping the bisulfite‐converted reads of that specific individual using bismark (Fig. 2c). The bismark pipeline was run as follows: for each individual, the *Bismark_genome_preparation* script was run on each of the two references in order to prepare the sets of indexes taking into account bisulfite conversion. Then, bisulfite‐converted reads of each individual were mapped to both references of that individual separately with bismark *,* choosing bowtie as the aligner module. In the case of *Heliosperma*, bowtie was called allowing for one mismatch between the reference and the query sequence, as we were mapping to all the alleles in that individual. In the case of *Dactylorhiza*, we used a maximum of two alleles per locus, although theoretically they could have up to four. RADseq analyses of the diploid progenitors have indicated that ~90% of the RAD loci in the *Dactylorhiza* allopolyploids originated from only one of the homeologs, and should therefore behave as ‘diploid’ loci (O. Paun, unpublished). However, to accommodate for this simplification, we aligned the query reads to the reference allowing for up to three mismatches. The next step was to extract the methylation information of each cytosine position using the *Bismark_methylation_extractor* routine (Fig. 2d) ignoring the first 4 bp in the reads, merging all non‐CpG cytosines, and directly producing a global cytosine report (CX_report files in the bismark output) for each individual sample with information on all cytosine contexts and all strands (original and complementary to the original, e.g. ‘+’ and ‘−‘). As we produced the reduced reference genome and the converted reads through the same protocol, we were able to easily recognize the position of each cytosine strandwise. According to our expectations, most of the reads corresponded to sequences complementary to the bottom strand and were therefore marked with a ‘−‘ in the report file. Due to a much lower coverage, we removed all cytosine positions identified on the ‘+’ strand. As the order of the loci was the same in the reduced references for all individual samples, all cytosines were uniquely identified by their position and thus comparable across samples.

An advantage inherent to the approach we developed for species without a reference genome available is that we automatically incorporate, and take into account, the genetic differences among analysed individuals. As we mapped the bisulfite‐converted reads to the actual sequences of each individual sample, we could clearly identify methylated Cs (as Ts in the converted sequences) without the risk of misidentifying C → T SNPs as SMPs (single methylation polymorphism; Schmitz *et al*. 2013). We produced summary tables indexed by the unique list of cytosine position including the number of reads covering each position, the number of reads supporting methylation at that position and the context of that position in each individual samples. We then compared individuals and identified methylation differences (i.e. a cytosine present in the reference sequences of all genotyped individuals) and nucleotide differences (i.e. cytosine position present only in some of the genotyped individuals). In addition, the context in which a position was found in each individual was also compared to identify changes of context due to neighbouring SNPs. This can be considered as a significant advantage over mapping bisulfite‐converted reads to a published reference genome (as in our stickleback experiment), where the amount of C → T SNPs between the samples and the reference, incorrectly identified by bismark as nonmethylated cytosines, is unknown. Our approach also allowed us to check whether the change in methylation state was due to a change in the sequence context of the selected cytosine (context swap). This is an important information as different molecular mechanisms are responsible for maintaining the methylation state through DNA replication according to the different sequence context (CpG, CHG and CHH), making the context critical for the maintenance of the methylation (see Introduction). Thus, when a base substitution occurs in the flanking region of a methylated cytosine, the sequence context can change (e.g. TC^met^GTTT: CpG context; TC^met^ATTT: CHH context) and the cytosine is expected to lose its methylation status. In such a case, the difference in methylation has a clear genetic determinant in the neighbouring sequence.

### Summary statistics of methylation patterns

The output of the bismark pipeline is a report file for each sample listing all cytosine positions analysed along the reference genome with information about coverage, methylation state and context (Fig. 2d). Only the CpG context was analysed in the stickleback experiment, whereas a summary of all three contexts was produced for the *Heliosperma* and *Dactylorhiza* experiments. We tested the performance of the proposed approach, considering both the wet‐lab and bioinformatic processing, by analysing nine additional samples (produced in a larger experiment on the *Heliosperma* species – data unpublished) with two technical replicates each. The methylation level (number of reads supporting methylation/number of all reads covering that position) was compared for all cytosine positions sequenced in both replicates for each sample. If the two technical replicates showed at a cytosine position a methylation level within 10% of each other, the signal was considered as reproducible.

Concerning each experiment separately, we used a python script (available as Supporting Information) to fetch the information from the report files produced by the bismark pipeline into three databases with positions as rows and individuals as columns. We recorded the depth of sequencing (coverage) in the first database, the methylation level calculated as the number of reads supporting methylation at that position divided by the coverage in the second database, and the cytosine context in the third database. Positions that were polymorphic in DNA sequence (i.e. SNPs) were recorded as missing data in those individuals without a cytosine in that position, or as positions with multiple contexts when the substitution involved a neighbouring nucleotide. In the case of *Heliosperma* and *Dactylorhiza*, each individual had two cytosine report files and hence, two entries (columns) in the summary databases. The summary databases were then used to analyse the differential level of methylation in selected groups after filtering the cytosine positions according to minimum coverage in each individual, minimum number of individuals per group and the context. Marine vs. freshwater fish were compared in the stickleback experiment, retaining only CpG positions covered in both individuals per group with coverage of at least 25 reads. *Heliosperma pusillum* and *H. veselskyi* were compared by selecting cytosine positions recorded in at least two individuals per group with coverage of at least 25 reads and analysing the three contexts separately. Finally, the two *Dactylorhiza* allotetraploids were compared by retaining cytosine positions called in at least one individual with coverage above 25 reads and analysing the three contexts separately.

Finally, statistical tests of methylation differentiation between groups on the positions passing the initial filters were performed. To reduce the number of comparisons, we excluded all positions showing across all individuals a mean level of methylation <10% or a standard deviation in the level of methylation <10%. We then applied a two‐sample Kolmogorov─Smirnov test as implemented in the *Scypy* python library (function *scipy.stats.ks_2samp*) obtaining a *P*‐value for each position, and we transformed the *P*‐values into *q*‐values (Storey & Tibshirani 2003), thus obtaining the False Discovery Rate associated with each comparison. In the case of *Heliosperma* and *Dactylorhiza*, when there was at least one cytosine position with *P*‐values <0.05, we extracted the locus sequence from the reduced reference genome and we performed a blast search using the nucleotide database in genbank. Blasting and annotation of *Heliosperma* loci were refined using the *Silene vulgaris* reference transcriptome (http://silenegenomics.biology.virginia.edu/; Sloan *et al*. 2012) recording the annotation and GO terms of all successful hits. In the case of the stickleback, cytosine positions showing methylation differentiation above 95% were annotated using the ensembl Variant Effect Predictor webtool (http://www.ensembl.org/info/docs/tools/vep/index.html). In the case of the fish and the tetraploid plant, where only four individual samples each were analysed (two per ecotype/species), the statistical assessment of methylation difference is proposed as a mere proof‐of‐concept.

## Results

### Conversion and sequencing efficiency

All our sequenced libraries appeared consistently converted by the sodium bisulfite treatment. The maximum frequency of methylation in a CHH context ranged from 2.3% in the *Dactylorhiza* experiment to 0.7% in the stickleback experiment. CHH methylation is assumed to be very low in animals (Feng *et al*. 2010a), so the amount of unconverted CHHs in the stickleback can be considered as a good proxy of incomplete conversion after bisulfite exposure. In line with our expectations, we got mainly reads corresponding to the complementary sequence of the converted bottom strand of each RAD fragment (Fig. 1). A low number of reads of the converted top strand was also sequenced (~0.1–1%). Reads of the converted top strand likely correspond to the initial template DNA that is retained after conversion and then directly ligated and sequenced on the flow‐cell. In the *Heliosperma* and *Dactylorhiza* experiments, cytosines scored on the top strand (marked with a ‘+’ in the bismark output) were discarded after the bismark methylation extraction step. In the stickleback experiment, we discarded all reads identified as OT (original top strand) and OB (original bottom strand) before building each individual cytosine methylation report. Using the nine technical replicates in *Heliosperma*, we were able to assess the reproducibility of the methylation level in 1642–5151 cytosine positions, depending on the individual replicate. Despite the low coverage of one set of replicates, reproducibility of cytosine methylation was very high, ranging from 93% to 95% (Table S2, Supporting information).

### Application in an animal system: freshwater vs. marine stickleback

**Figure 3:**
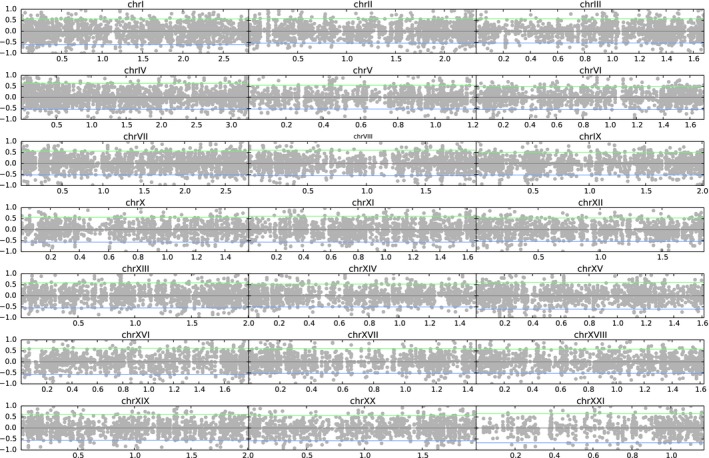
Differential methylation between the two‐three‐spine stickleback ecotypes in the CpG context, separately given for the 21 chromosomes. A value of 0 indicates equal methylation between the two groups. A value of 1 indicates complete methylation in the freshwater ecotype and complete lack of methylation in the marine ecotype, while a value of −1 indicates the opposite. Above the green line and below the blue line is the top 2.5% of the cytosine positions that are differentially methylated. The methylation differences in the remaining contigs in the reference linkage group called ‘ChrUn’ are shown in Fig. S1, Supporting information. Genomic locations and annotations of differentially methylated cytosine positions are given in Table S3 (Supporting information).

In this experiment, the average number of bisulfite converted reads per sample was *c*. 14 000 000, which mapped on an average length per sample of 2.2 Mbp in the reference genome. The mapping resulted in an average coverage of 480× and an average mapping efficiency across individuals of 79% (Table S1, Supporting information). The average methylation level, as estimated in the bismark methylation extractor step, for the cytosines in a CpG context was 68.5%. In addition, a few Cs in CHG (0.5%) and CHH (0.7%) contexts appeared also as methylated, but these were discarded from further analyses. The methylation level was scored across the four individuals at 49 477 CpG positions along the stickleback genome (Fig. 3; Fig. S1, Supporting information); 29 953 positions had a mean methylation across all samples >10% and a standard deviation >10% (Table S3, Supporting information). A total of 155 cytosine positions were consistently methylated in both individuals of either the freshwater or the marine population (methylation difference between the two populations above 95%). However, because of the low sample size (two individuals per group), *P*‐value and *q*‐value at these positions were not significant. Genomic locations for all differentially methylated positions and ensembl annotations for positions showing a difference in methylation above 95% are indicated in Fig. 3 and in Table S3, (Supporting information).

### Application in a diploid plant system: Heliosperma pusillum vs. H. veselskyi

**Figure 4:**
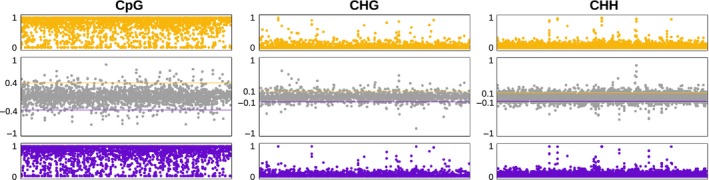
Differential methylation between the two *Heliosperma* species, given separately for each methylation context. 2855, 5555 and 18 427 cytosine positions were assessed in the CpG, CHG and CHH contexts respectively. The top panels (orange) show the average methylation of *H. pusillum*, the bottom panels (blue) show the average methylation of *H. veselskyi*. The middle panels (grey) show the methylation difference between the two species, where a value of 1 indicates complete methylation in *H. pusillum* and complete lack of methylation in *H. veselskyi*, while a value of −1 indicates the opposite. The lines in the middle panels indicate the 95% quantile. The annotations of differentially methylated cytosine positions are given in Table S4 (Supporting information).

The mean efficiency of mapping to the reduced reference genome built by concatenating ~1710 RADseq fragments, each 94 base pairs long, was 21.5% with an average number of mapping reads per sample of ~617 000 (Table S1, Supporting information). The lower mapping efficiency recorded in the experiments without a whole‐genome reference is a consequence of the selection of loci applied when assembling the reduced‐representation genome. Across all samples, the average methylation of the screened cytosines in CpG, CHG and CHH context was 74%, 1.8% and 1.1% respectively, as in the bismark output summary. After filtering for a minimum number of two individuals per group and minimum coverage of 25×, and excluding all positions where a context change occurred in at least one individual, we compared the average methylation level by position between *H. pusillum* and *H. veselskyi* in 2855 CpG, 5555 CHG and 18 427 CHH positions (Fig. 4). Generally, we found a conserved pattern of methylation across all samples, independent of the native habitat type. A slightly higher variance in the methylation level was found in the CpG methylation, probably due to the globally higher methylation level in this context. Several outlier positions showing a signal of differential methylation between the two ecotypes can be identified in this context. We compared the distributions of the methylation as recorded across all individuals in each group by a two‐sample Kolmogorov─Smirnov test. A total of 212 cytosine positions occurring on 156 RAD loci, across all contexts, showed a *P*‐value <0.05 and 17 positions, occurring in 15 different RAD loci, showed a *q*‐value <0.05. Several cytosine positions cooccurring on the same locus showed a similar significant signal of divergence (Table S4, Supporting information). Blasting each locus sequence to the NCBI *nt* database and to the *Silene vulgaris* transcriptome, it was possible to annotate 72 of the 156 loci including cytosine positions with significant *P*‐value and 6 of the 15 loci including positions with significant *q*‐values. Considering the latter case, among the suggested genes we found a galactose‐oxidase and a few proteins with binding or catalytic activity (Table S4, Supporting information). The high proportion of positions mapping to genes might be due to the general enrichment in coding regions produced by the RADseq restriction enzyme (restriction site with 75% GC content).

### Application in a tetraploid plant system: Dactylorhiza majalis vs. D. traunsteineri

**Figure 5:**
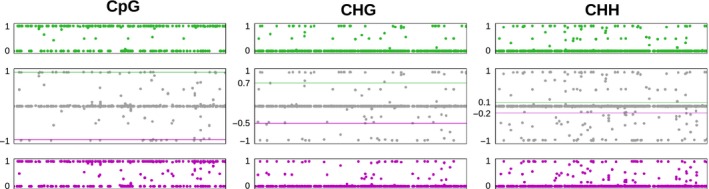
Differential methylation between the two *Dactylorhiza* tetraploids, given separately for each methylation context. 705, 1153 and 3533 cytosine positions were assessed in the CpG, CHG and CHH context respectively. The top panels (green) show the average methylation of *D. majalis*, the bottom panels (purple) show the average methylation of *D. traunsteineri*. The middle panels (grey) show the methylation difference between the two species, where a value of 1 indicates complete methylation in *D. majalis* and complete lack of methylation in *D. traunsteineri*, while a value of −1 indicates the opposite. The lines in the middle panels indicate 95% quantiles. The annotations of differentially methylated cytosine positions are given in Table S5 (Supporting information).

We also tested the applicability of our approach for the polyploid species, performing a test on four individuals of two sibling allotetraploid orchids. As in the *Heliosperma* experiment, we mapped the bisulfite‐converted reads to a reduced reference genome built using a standard RADseq data set of 3180 loci, 94 base‐pair long each, including only two random alleles per locus per individual in order to simplify downstream analyses. Average mapping efficiency was 20% with ~534 000 mapping reads per sample and the average methylation level was 49% in CpG, 3.7% in CHG, and 2% in CHH context as summarized in the bismark output files (Table S1, Supporting information). The overall pattern of methylation between the two tetraploid species showed clear outliers standing out from a baseline of positions with a similar level of methylation (Fig. 5). Comparing the level of methylation over 705 CpG positions genotyped in at least one population of each polyploid, we found that 65 of them were fully methylated in one species and unmethylated in the other. Very few cytosines showed an intermediate level of methylation, in particular in *D. majalis* (Fig. 5). We screened also 1153 CHG positions and 3533 CHH positions of which 77 and 135 respectively, showed a methylation level above 50% in one or the other species and 53 and 103 respectively, were differentially methylated between the two species. The statistical test of significance for the difference in the methylation level of each cytosine (including an FDR correction at *q*‐value <0.05) highlighted ten cytosine positions, whereas 135 cytosine positions showed a *P*‐value <0.05 of which 66 produced significant hits to the nucleotide database in genbank (Table S5, Supporting information). However, given the very low sample size in this experiment, these results should be considered as a mere proof‐of‐concept and need to be validated by a larger data set.

## Discussion

### Consistent methylation patterns across ecotypes

Significant gene expression differences, and therefore divergent regulatory landscapes, are expected between individuals occupying distinct ecological niches, even if they are closely related (Flatscher *et al*. 2012). In addition, continuous exposure to specific abiotic and biotic conditions should shape distinct heritable epigenetic landscapes (Bossdorf *et al*. 2008; Jablonka & Raz 2009; Feil & Fraga 2012) through differential selection of stochastically originated epialleles. Lastly, the potentially reversible nature of epigenetic patterns implies that drift may affect them faster than genetic patterns. Therefore, major differences in the epigenetic landscape are expected when comparing distinct ecotypes. Indeed, several MSAP studies identified extensive DNA methylation differentiation between natural populations from distinct environments, exceeding genetic differentiation (e.g. Lira‐Medeiros *et al*. 2010; Medrano *et al*. 2014; Schultz *et al*. 2014).

Using our bsRADseq approach, we investigated the epigenetic differentiation between populations with divergent ecological and morphological characteristics in three different biological systems. Our analyses point to a general stability across ecological ranges of the level and distribution of DNA methylation. Given that the environment can trigger significant methylation alterations (e.g. Dowen *et al*. 2012), our finding is particularly interesting considering that we investigated individuals from natural populations.

Even if our approach is not specifically designed to enrich for CpG‐rich islands, as no CpG is present in the restriction site of the enzyme used, higher representation of genomic regions rich in Gs and Cs was expected as we used an enzyme with high GC content in the recognition site (75%). It is therefore possible that our analyses mainly represent patterns in a portion of the genome that may be particularly epigenetically stable. However, this stability implies a constitutive nature of epigenetic signals, or that adaptation through epigenetic effects is achievable through alterations at only a limited number of loci. Repeating the experiment using an enzyme with a different CG content in the restriction site would help in better understanding the generality of the pattern observed here across the whole genome. However, similar findings have recently been reported in near‐isogenic lines of *Arabidopsis thaliana* that have diverged under natural conditions for at least 100 generations (Hagmann *et al*. 2015), with only minor environmentally related epigenetic variation identified. Further, a study of the epigenetic differentiation along the northward range expansion of *Taraxacum officinale* in Europe reports only modest heritable differentiation between samples thousands of km apart along a latitudinal gradient (Preite *et al*. 2015).

The epigenetic stability that we find between individuals growing in different environments is evident in diploid *Heliosperma,* but also in stickleback and tetraploid *Dactylorhiza*. The Heliosperma study system is particularly interesting as the two populations compared here belong to two species that are hypothesized to have recurrently originated from each other at several locations in Eastern Alps (Frajman *et al*. 2009; E. Trucchi, B. Frajman, T. Haverkamp, P. Schönswetter & O. Paun, unpublished). Such a stability of DNA methylation patterns across individuals that are adapted to contrasting environments may indicate that ecological adaptation, at least in its early phases, does not require extensive epigenetic divergence, but may be related to few outlier (epi)loci. Interestingly, the signal of differential methylation between the two species is often consistent across the cytosine positions cooccurring on the same locus: nine out of the 15 loci, each 94 base‐pair long and containing differentially methylated positions with a *q*‐value <0.05, contain more than one differentially methylated position (considering all positions with *P*‐value <0.05). In several cases, the differential methylation is also consistent across different contexts. These loci may then overlap with differentially methylated regions, usually 10–1000 base‐pair long, that have been suggested to be functionally linked to gene expression, exon─intron boundaries definition and TEs silencing (Chodavarapu *et al*. 2010; Becker *et al*. 2011; Schmitz & Zhang 2011). Our statistical test identified potential candidates with functions related to binding and catalytic activity. Additional studies should confirm the significance of these loci across a larger sampling. Finally, it should be noted that the overall degree of methylation in CpG context scored in *Heliosperma* (~75%) is extremely high in comparison with previously studied *Arabidopsis thaliana* and *Oryza sativa* (Feng *et al*. 2010a), whereas the CHG and CHH methylation is comparatively rather low.

In case of the polyploids, a broad array of stochastic genomic responses is expected after whole‐genome doubling and hybridization, with natural selection sorting this variation according to the environment in which the new lineage forms (Madlung & Wendel 2013). As the sibling allopolyploids studied here formed independently in time and space, and are adapted to distinct environments (Pillon *et al*. 2007; Paun *et al*. 2010, 2011), their overall epigenetic landscape is expected to be distinct from one another. In addition, the presence of duplicated genes renders the possibility of a much greater variation, and therefore differentiation, due to alternative silencing of homeologs via epigenetic regulation. Yet in tetraploid *Dactylorhiza,* we recorded a similar methylation profile with <10% of the cytosines being fully methylated in one ecotype and unmethylated in the other over the three possible contexts. A similar degree of differentiation was also recorded with MSAP using a larger sample size covering the entire distribution of these polyploids (Paun *et al*. 2010).

The result is less clear in the case of the stickleback where we find a higher variation in the methylation profile between the two ecotypes. One possible explanation is that we did not produce a specific RADseq reference for each individual stickleback analysed, but rather mapped the bisulfite‐converted reads onto a reference genome, thereby not taking into account the possible genetic differences between the reference genome and the analysed individuals. It is not clear how much bias in scoring the methylation level was introduced at that stage. More importantly, methylation stability across ecotypes, and in general among individuals, is expected to be different in animals and plants (Feng *et al*. 2010b). Nevertheless, we were able to identify several cytosines exhibiting divergent patterns of methylation between the two groups of samples from different habitats (Fig. 3, Fig. S3, Table S3, Supporting information). On a global scale, the methylation level recorded in this system (68.5% in CpG context, and very low in the other contexts) is in line with what was found previously in the stickleback (Smith *et al*. 2015) and in other vertebrates (Feng *et al*. 2010a). The genetic basis of the recurrent adaptation to freshwater from the marine environment in the three‐spine stickleback is well understood (Colosimo *et al*. 2004; Jones *et al*. 2012; O'Brown *et al*. 2015). However, epigenetic marks are expected to play an important role in controlling both gene expression and regulatory pathways (Jaenisch & Bird 2003). Extending this analysis to a larger number of populations and including replicate samples from different tissues will help elucidate which of these divergent epiloci are characteristic of the overall differentiation between marine and freshwater stickleback ecotypes.

### Advantages and limits of bsRADseq

Several methods have been developed to explore genome‐wide methylation patterns (Lister & Ecker 2009). Some of these methods do not apply any bisulfite‐conversion of the DNA, but rather sequence only hyper‐ or hypo‐methylated regions of the genome after a selection step mediated by immunoprecipitation (MeDIP; Weber *et al*. 2005) or enzymatic affinity (Bio‐CAP; Blackledge *et al*. 2012). In other approaches, sodium bisulfite treatment is followed by whole‐genome sequencing (BS‐Seq; Cokus *et al*. 2008; Lister *et al*. 2009). It is also possible to first reduce the complexity of the genome using a restriction enzyme that can be either methylation sensitive or not, and follow this with bisulfite treatment and sequencing (e.g. reduced representation bisulfite sequencing, RRBS; Meissner *et al*. 2005). The targets of this kind of selection are usually CpG‐rich islands (e.g. Smith *et al*. 2015), which support the implementation of RRBS in biological systems where CpG‐rich islands are a common feature of the genome, such as vertebrates (Feng *et al*. 2010a). However, RRBS has also been recently applied in a plant species without a reference sequence nor any prior knowledge on genome architecture (Platt *et al*. 2015). Nonetheless, all these methodologies, including bsRADseq, can fully take advantage of a reference genome, when available, in order to properly assess the general genomic context of methylation.

The approach tested here, bsRADseq, couples the reliability of bisulfite conversion (Frommer *et al*. 1992) and the flexibility of RAD sequencing (Baird *et al*. 2008) and is thus particularly suited to screen medium to large‐sized genomes across many individuals. A standard RADseq reduced‐representation genome may then be used as a reference. The modular aspect of the proposed method makes it flexible. In the test experiments presented here, we screened between 160 000 and 300 000 base pairs of the genomes of plant species for which no genome reference is available. However, the number of sequenced loci in each experiment is governed by the choice of the restriction enzyme, which allows for fine‐tuning the desired representation of the genome, but also the plexity for each library (Davey & Blaxter 2010). The analysis of the methylation pattern in a reduced portion of the genome allows for screening many individuals in a cost‐effective way, enabling population‐level analyses of epigenetic signals in non‐model species. As compared to a standard RADseq approach, the initial cost of ordering methylated adapters for the library preparation is high. Using a short version of the P1 adapter to be extended in the final PCR steps and implementing a combinatorial indexing system as we did in the presented experiment would, however, limit the initial investment. Nevertheless, given the low amount of barcoded P1 adapter needed per sample in each library, this initial cost can be spread over several hundreds of libraries making it possible to share resources across labs.

On the other hand, as bsRAD loci are relatively short (100–150 bp single‐read, up to 500–750 bp when sequencing paired‐end), it may be difficult to identify relevant differentially methylated regions (DMRs) that could be longer than a single‐read RAD locus (Lister & Ecker 2009; Lister *et al*. 2009). It is, however, possible to extend the RAD loci up to 750 bp by assembling the paired‐end reads into mini‐contigs. When a reference genome is available, the two sides of each restriction site, which are both sequenced in the RADseq protocol (Baird *et al*. 2008), can be merged, resulting in ~1.5 kb of contiguous sequence. When a reference genome or a genetic map is available, the information from loci in close linkage could be summed up over windows, in particular if opting for an increased density of the genomic screening by using a frequently cutting restriction enzyme. This, of course, comes at the cost of decreasing sample plexity per sequencing run. Questions concerning the average length of DMRs, and/or how many differentially methylated cytosines are needed to statistically call a DMR, are still open, and might differ depending on the biological system studied. In addition, the degree of methylation differs among organisms: for example, whereas plants usually present a mosaic DNA methylation pattern, vertebrates are characterized by a more stable global DNA methylation pattern (Suzuki & Bird 2008). Even if differentiation in the methylation pattern can be restricted to some well‐defined and long genomic islands in vertebrates (Long *et al*. 2013), a neutrality test based on the frequency spectra of each single methylation polymorphisms (SMPs) was recently proposed (Wang & Fan 2015).

### Identifying accurate methylation polymorphisms

The information provided by the standard RAD sequencing data set in both the *Heliosperma* and *Dactylorhiza* experiments allowed us to remove all cytosine positions from the analyses that were genetic variants in only some of the individuals, or where a SNP occurred in the flanking 2 base‐pairs downstream region, thereby changing the methylation context. Taking into account SNPs when extracting methylation information is particularly important when the reference genome was sequenced from a phylogenetically distant population. In such cases, C → T substitutions between the reference genome and the analysed sample may be incorrectly recorded as unmethylated cytosines. In addition, a change of context (e.g. from CpG to CHH) due to substitutions will likely trigger a methylation difference, which should not be considered as epigenetic divergence. An analysis of a larger data set of the two *Heliosperma* ecotypes, using a consensus RAD reference, where we fixed the most common allele across all individuals (E. Trucchi, B. Frajman, T. Haverkamp, P. Schönswetter & O. Paun, unpublished), revealed that: (i) 3.8% of the SNPs that were a mutation from A,T, or G to a C, would have resulted as positions not scored in some individuals, (ii) 1.6% were changes from C to A or G that would have resulted in missing data in some individuals and (iii) 2.4% were C to T changes that would have been scored incorrectly as a differentially methylated position in some individuals. In addition, we recorded that: (i) 1.4% of the C positions experienced a context change between CpG and CHH in at least one individual, (ii) 0.3% between CpG and CHG, (iii) 1.8% between CHG and CHH and (iv) 0.02% among CpG, CHG and CHH. When a position was found in different contexts across the individuals screened, its methylation level was often coherent with the average methylation level of the context. In other words, the same cytosine found in CpG context in some individuals showed a generally higher level of methylation than when it was found in CHH or CHG contexts in other individuals. When comparing large samples or distantly related populations, genetic variation might produce a non‐negligible bias in scoring methylation differentiation. A SNP matrix can be produced from all (or a representative subset of) individuals, and then included in the analysis, masking the variable positions. With respect to this issue, we have shown that bsRADseq can be easily coupled with standard RADseq analysis to obtain information about the genetic background of each individual and overcome these potential problems.

## Concluding remarks

Describing the extent and type of differentiation in the DNA methylation profile across ecological ranges is fundamentally important for understanding the role of gene regulation in adaptation. We showed here that differences in the methylation pattern clearly stand out over a background of generally low level of epigenetic differentiation. Whether this is unique to the natural systems studied here or rather a general rule can only be answered by increasing the number of natural systems screened. Our work introduces the use of bsRADseq as a cost‐effective and scalable tool to study the epigenetic differentiation between ecologically divergent populations of model and non‐model species, and we hope it will stimulate more work on these topics.

E.T. designed the methodological approach; E.T., A.B.M. and O.P. designed research; E.T., A.B.M. and G.D.G implemented the method; E.T., A.B.M., M.T.L. and O.P. performed the lab work; E.T. performed research, contributed new analytical tools, and analysed data; E.T., A.B.M., G.D.G, P.S. and O.P. wrote the paper.

## Data accessibility

Both standard and bisulfite‐converted raw reads data are publicly available on the SRA archive (Acc. num. SRP065672, SRP065676). Reduced‐representation reference genome sequences of *Heliosperma* and *Dactylorhiza*, and all report files produced by the bismark pipeline are available on Dryad (doi:10.5061/dryad.hr0qt). Supplementary figures and tables and custom python scripts used in the analyses are available as Supporting Information.

## Supporting information


**Fig. S1** Differential methylation between the two‐three‐spine stickleback ecotypes in the CpG context, on ‘Unknown’ linkage group.

**Table S1** Samples information, SRA accessions of standard and bisulfite‐converted reads and summary statistics of bismark mapping.

**Table S2** Replicated samples analysis.

**Table S3** Differentially methylated positions in the stickleback experiment. Annotation is provided for positions showing more than 95% difference between the two groups.

**Table S4** Differentially methylated positions (*P*‐value < 0.05) in *Heliosperma* experiment. Positions showing significant *q*‐values (<0.05) are in bold.

**Table S5** Differentially methylated positions (*P*‐value < 0.05) in *Dactylorhiza* experiment. Positions showing significant *q*‐values (<0.05) are in bold.

**Appendix S1** Custom python script used to summarize the output of bismark (CX_reports).

**Appendix S2** Custom python script used to test the statistical significance of differentially methylated positions.
